# Inhibition of Epstein-Barr virus reactivation by the flavonoid apigenin

**DOI:** 10.1186/s12929-016-0313-9

**Published:** 2017-01-05

**Authors:** Chung-Chun Wu, Chih-Yeu Fang, Yu-Jhen Cheng, Hui-Yu Hsu, Sheng-Ping Chou, Sheng-Yen Huang, Ching-Hwa Tsai, Jen-Yang Chen

**Affiliations:** 1 National Institute of Cancer Research, National Health Research Institutes, No.35, Keyan Road, Zhunan Miaoli, Miaoli County Taiwan; 2 Department of Pathology, Wan Fang Hospital, Taipei Medical University, Taipei, 116 Taiwan; 3 Department of Microbiology, College of Medicine National Health Research Institutes, National Taiwan University, No.35, Keyan Road, Zhunan Town, Miaoli County, Taipei Taiwan

**Keywords:** Epstein-Barr virus, Apigenin, Reactivation, Nasopharyngeal carcinoma

## Abstract

**Background:**

Lytic reactivation of EBV has been reported to play an important role in human diseases, including NPC carcinogenesis. Inhibition of EBV reactivation is considered to be of great benefit in the treatment of virus-associated diseases. For this purpose, we screened for inhibitory compounds and found that apigenin, a flavonoid, seemed to have the ability to inhibit EBV reactivation.

**Methods:**

We performed western blotting, immunofluorescence and luciferase analyses to determine whether apigenin has anti-EBV activity.

**Results:**

Apigenin inhibited expression of the EBV lytic proteins, Zta, Rta, EAD and DNase in epithelial and B cells. It also reduced the number of EBV-reactivating cells detectable by immunofluorescence analysis. In addition, apigenin has been found to reduce dramatically the production of EBV virions. Luciferase reporter analysis was performed to determine the mechanism by which apigenin inhibits EBV reactivation: apigenin suppressed the activity of the immediate-early (IE) gene Zta and Rta promoters, suggesting it can block initiation of the EBV lytic cycle.

**Conclusion:**

Taken together, apigenin inhibits EBV reactivation by suppressing the promoter activities of two viral IE genes, suggesting apigenin is a potential dietary compound for prevention of EBV reactivation.

**Electronic supplementary material:**

The online version of this article (doi:10.1186/s12929-016-0313-9) contains supplementary material, which is available to authorized users.

## Background

Epstein-Barr virus, a member of the γ-herpesviruses, infects most of the human population worldwide [1]. It plays a causative role in infectious mononucleosis, hairy leukoplakia, and post-transplant lymphoproliferative disorder [1] and is highly associated with several human malignancies, including Burkitt’s lymphoma (BL) and nasopharyngeal carcinoma (NPC). EBV mainly infects human circulating B cells and is maintained in a latent state. Upon stimulation by chemical agents, e.g. 12-o-tetradecanoyl-phorbol-1,3-acetate (TPA) and sodium butyrate (SB), human IgG or cytokines, EBV enters the lytic stage. It sequentially expresses immediate early (IE), early (E) and late (L) proteins and, eventually, mature virions are released [1].

In the recent decade, increasing evidence has suggested that EBV lytic reactivation plays an important role in various human malignancies. In seroepidemiological studies, elevation of antibody titers against EBV lytic proteins in NPC and BL patients has suggested that EBV reactivation is highly correlated with cancer progression, poor prognosis and tumor recurrence of NPC [2–4]. Retrospective studies revealed that NPC patients have elevated antibody titers against EBV lytic antigens prior to diagnosis and prospective surveys have revealed that individuals with elevated antibody titers have a higher incidence of NPC [5–7]. Moreover, the proteins and mRNAs of EBV lytic genes were detectable in clinical samples from NPC patients [8–10]. Recently, we found that EBV reactivation induces genomic instability and enhances tumor progression [11, 12]. EBV lytic proteins, such as viral DNase, terminase and kinase, also have been shown to have the ability to induce genomic instability via different mechanisms [13–15]. These reports revealed that inhibition of EBV reactivation is beneficial for cancer prevention and therapy [16, 17]. Several types of compounds also have been developed for the inhibition of EBV reactivation: (i) Nucleoside analogs, which inhibit the EBV lytic cycle by blocking DNA replication, are used extensively in antiviral therapy (e.g. acyclovir, ACV, and ganciclovir, GCV) [18]. (ii) Non-nucleoside drugs have been developed to block EBV replication (e.g. maribavir) [19]. (iii) Dietary ingredients, e.g. retinoic acid, epigallocatechin gallate, curcumin and sulforaphane, also have been suggested to have the potential to inhibit the EBV lytic cycle [20–23]. Regarding clinical application, dietary compounds are attractive for the inhibition of EBV reactivation because of their safety and convenience. We screened several dietary compounds to identify those are able to inhibit the EBV lytic cycle and found that apigenin has the ability to inhibit the EBV lytic induction effectively.

Apigenin is a member of the flavonoids, which are present in abundance in common fruits and vegetables [24]. Apigenin has anti-oxidative, anti-mutagenic, anti-carcinogenic, anti-inflammatory, anti-proliferative and anti-progressional properties [24]. However, the association between these biological functions and, the anti-viral effect of apigenin is less well understood.

In this study, we found apigenin inhibits EBV reactivation into the lytic cycle and virion production by EBV-positive NPC cells. Moreover, we addressed the question whether apigenin represses the promoter activities of the EBV IE genes, Zta and Rta, to explore the possible mechanism of this inhibitory effect. This study gives new insight into the biological application of apigenin and provides an alternative choice for anti-EBV therapy.

## Methods

### Compounds and antibodies

Apigenin and the induction agents, TPA, SB, TSA, SAHA and romidepsin were purchased from Sigma-Aldrich Co. Antibodies used in western blotting and immunofluorescence analysis include anti-EBV Rta 467 (unpublished), anti-BMRF1 (EAD) 88A9 [25], anti-EBV Zta 4 F10, anti-DNase 311H [26], and anti-β-actin (Sigma-Aldrich Co.).

### Cell lines

NA and HA cells, are EBV converted cells obtained by co-culture of rAkata cells with TW01 and HONE-1 cells, respectively, and were selected by G418 (Sigma-Aldrich Co) treatment [27]. All epithelial cell lines were maintained in DMEM (Dulbecco’s modified Eagle’s medium) supplemented with 10% fetal calf serum (FCS). P3HR1 [28], an EBV-positive Burkitt’s lymphoma cell line, was maintained in RPMI-1640 supplemented with 10% FCS.

### Cytotoxicity assay

The cytotoxicity of apigenin to each cell line was determined by WST-1 assay (Invitrogen) according to the manufacturer’s instructions. The half maximum cytotoxic concentration (CC_50_) for each cell line was the concentration of apigenin which killed 50% of the cells. The results were averaged from at least three independent experiments to calculate the mean and standard deviation.

### Western blotting analysis

Western blotting has been described in our previous report [23]. Briefly, the samples were subjected to SDS-PAGE and then transferred to Hybond-C membranes (Amersham Biosciences Ltd.). After blocking for 1 h, the membranes were incubated with the antibodies indicated for 24 h at room temperature and then washed three times with washing buffer (10 mM Tris–HCl, pH 8.0, 0.9% NaCl). The blots were then treated with horseradish peroxidase-labelled goat anti-mouse IgG (Amersham Biosciences Ltd., diluted 1:20,000) for 1 h at room temperature. After washing three times, the blots were developed with freshly prepared substrate (Amersham Biosciences Ltd.). The luminescence was detected by a short exposure to x-ray film.

### Immunofluorescence staining

The cells indicated were seeded on cover slides for 24 h and then treated with various concentrations of apigenin for 25 h, or pretreated with apigenin for 1 h followed by TPA (40 ng/ml) and SB (3 mM) treatment for a further 24 h. The cells then fixed with 2% formaldehyde for 10 min and permeabilized with 0.4% Triton X-100 in PBS for a further 5 min. After washing three times, the cells were blocked in 4% FCS in PBS for 30 min. The cells were treated with anti-EAD antibody which was diluted in 1:10 at 37 °C for 1 h. After washing, rhodamine-conjugated goat anti-mouse IgG, diluted 1:100 in 4% FCS-PBS was added. After incubation with secondary antibody at 37 °C for 1 h, the cells were washed and observed by fluorescence microscopy. The nuclei were visualized by DAPI (Sigma-Aldrich Co) staining.

### Determination of the copy number of the viral genome

The procedures for determination of viral genome copy numbers were derived from a published paper [23]. Briefly, EBV-positive NPC NA cells (1 × 10^6^ cells/well) were incubated with TPA (40 ng/ml) and SB (3 mM) for 48 h after pre-treatment of apigenin for 1 h. The supernatants were harvested and filtered through a 0.45 μM filter, then each sample was incubated with DNase I and 10× DNase I buffer (10 mM Tris–HCl, 2.5 mM MgCl_2,_ 0.5 mM CaCl_2_,and pH 7.6) at 37 °C for 60 min, then 2 mM EDTA (pH 8.0) was added to inhibit DNase I activity. Each sample was then treated with 0.1 mg/ml proteinase K (1:1 [vol/vol]) at 50 °C for 60 min and the reactions were stopped at 75 °C for 20 min. Subsequently, each sample and standards (1 μl, see the description below) were examined for the BALF5 sequence (sense: 5′-CGGAGTTGTTATCAAAGAGGC-3′; antisense: 5′-CGAGAAAGACGGAGATGGC-3′), the DNA polymerase of EBV, by real-time quantitative PCR (qPCR) amplification [29]. The qPCR conditions were: 5 s denaturation at 95 °C, 20 s annealing at 60 °C and 2 s extension of primers at 72 °C for 45 cycles. The specificity of the PCR reaction was monitored by melting curve analysis (65–95 °C, 0.1 °C/s) in the LightCycler 480 (Roche Applied Science). The results from three independent experiments were used to calculate the mean and standard deviation.

### Plasmid constructions

The 5′-serial-deleted mutants of Zp were reported previously [30]. The mutants of each domain of Zp were constructed by means of PCR-based site-directed mutagenesis. The primers used referred to the following reports: mZIIIA (forward 5′-TAGAAACTATGCAGAATTCACAGGCATTGCTAA) [31]; mZIIIB (forward 5′-ATGAGCCACAGGATCCGCTAATGTACCTC) [31]; mZ1D-1 (forward 5′-ATGTACCTCATAGACAATACTAAATTTAGCACGTC) [30]; mZ1D-2 (forward 5′-TCATAGACACACCGCCATTTAGCACGTCC) [30]; mZII-1 (forward 5′-CACGTCCCAAACGAATTCATCACAGAGGA) [32]; ZII-2 (forward 5′-CAAACCATGACATGGATCCGGAGGCTGGTG) [32].

### Transfection and luciferase reporter activity analysis

The construction of the Zp and Rp reporter plasmids was described in a previous report [23]. Transfection procedure was carried out using Lipofectamine 2000 (Invitrogen) according to the manufacturer’s instructions. For Zp or Rp activation by TPA + SB, plasmid Zp or Rp was first transfected into NA and parental TW01 cells. After 3 h transfection, apigenin was added or not for pre-treatment for 1 h, and then TPA (40 ng/ml) and SB (3 mM) were added to induce EBV into the lytic cycle. For transfection of Zta-expressing plasmid plus Zp or Rp, Rta-expression plasmid plus Zp or Rp, cells (2 × 10^5^ cells/well) were seeded 24 h before transfection. Plasmid mixtures were transfected using the procedures described above. After induction for 24 h, cells were lysed in HEPES lysis solution and the lysates were subjected to luciferase activity assay (Promega). Each lysate sample was quantified for the expression of β-actin to control for variation in the amount of each sample (data not shown). For analysis of the activities of the Zp mutants, by comparison with mock treatment (M) of the vector control (V), for which the activity was set to 1, the relative activities are indicated as N fold induction over the activity of the vector control. The fold of inhibition is the induction folds of the TPA + SB plus apigenin (TS + A20 and TS + A50) groups compared to that of the TPA + SB (TS) group. The mean and standard deviation of each sample were calculated from at least two independent experiments in duplicate.

## Results

### The cytotoxicity of apigenin to epithelial cells

**Fig. 1:**
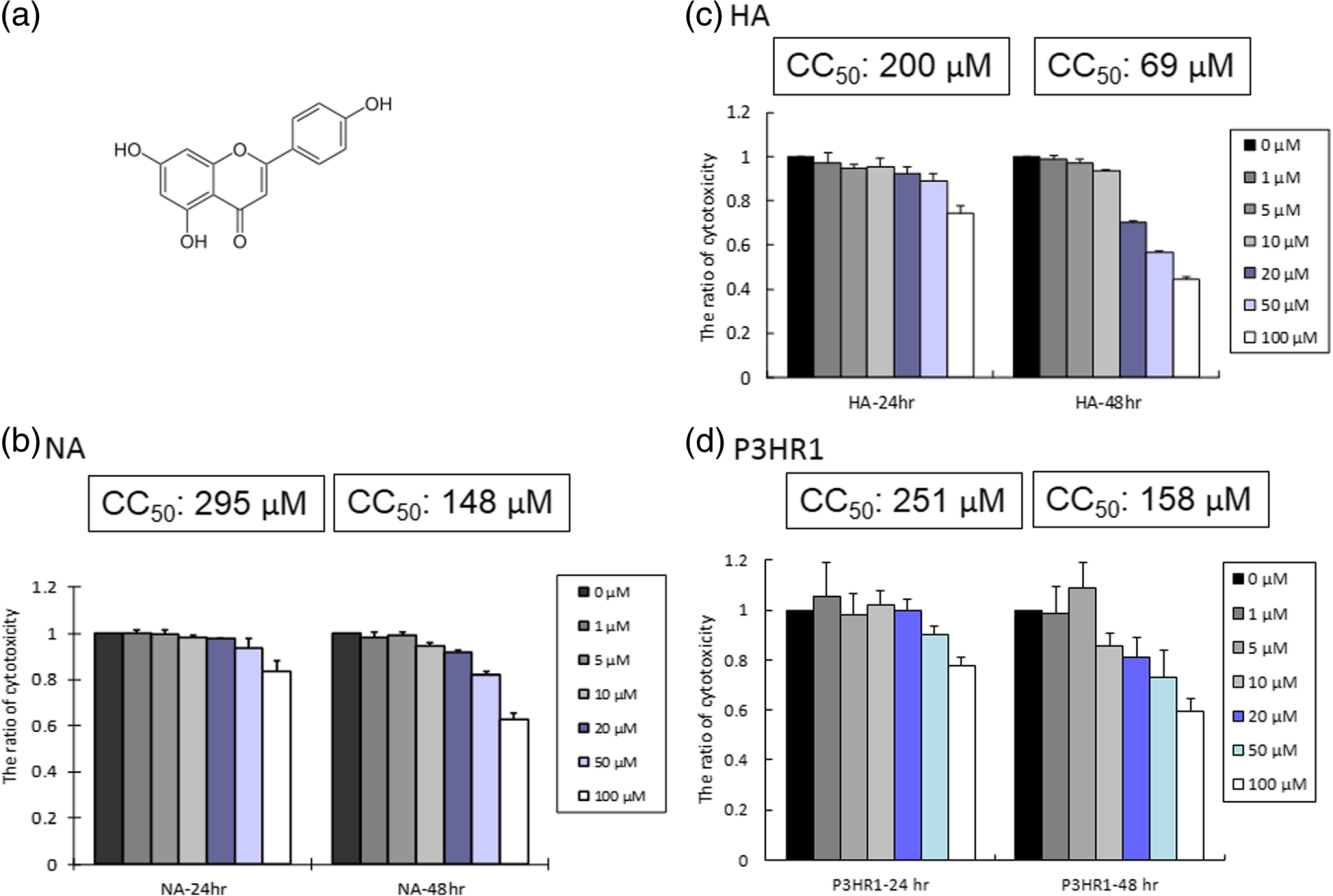
The cytotoxicity of apigenin to epithelial cells. (**a**) The chemical structure of apigenin. (**b**) NA, (**c**) HA and (**d**) P3HR1 cell lines were treated with apigenin for 24 h. Cell viability was determined by WST-1 assay, as described in Methods. The values are means ± SD from at least two separate experiments. CC_50_ values also were calculated and are given at the top of each group

Apigenin, which is known as 4’, 5, 7,-trihydroxyflavone (Figure 1a), is a flavonoid of the flavone structural type. In order to rule out the interference of toxic effects of apigenin treatment of the cells, we first tried to determine the cytotoxicity of apigenin to NPC cells. Two EBV-positive cell lines, NA and HA, were tested by WST-1 assay for susceptibility to apigenin cytotoxicity after 24 h and 48 h treatments. As shown in Figure 1b, apigenin had almost no effect on NA cells after 24 h, and had low cytotoxicity after 48 h. To the other NPC cells line, HA, apigenin also showed little cytotoxicity after 24 h, however, it had significant cytotoxicity with 20 ~ 100 μM for 48 h (Figure 1c). The cytotoxicity of apigenin to the B cell line P3HR1 was also determined (Figure 1d). To evaluate these data more precisely, the values of half maximum of cytotoxicity concentration 50 (CC_50_) were calculated and are shown at the top of Figures 1b to d. The CC_50_ values of all three cell lines for 24 h were greater than 200 μM, while the values for 48 h treatment of NA, HA and P3HR1 were 148, 69 and 158 μM (Figures 1b to d), respectively. To determine the cytotoxicity of the combination of apigenin and TPA + SB, NA, HA and P3HR1 cell lines were pre-treated with various concentrations of apigenin for 1 h and then TPA + SB were added. After 24 and 48 h incubation, the cells were subjected to WST-1 assay. The results indicated that apigenin combined with TPA + SB treatment did not cause severe cytotoxicity to the three cell lines (Additional file 1). Taken together, we determined that the CC_50_ values of apigenin are 200 to 295 μM and 69 to 158 μM for 24 and 48 h, respectively, which is similar to the effect on other cancer cells [33]. Thus we chose 1 ~ 50 μM as our working concentrations for further studies.

### Inhibition of expression of the EBV lytic proteins by apigenin

**Fig. 2:**
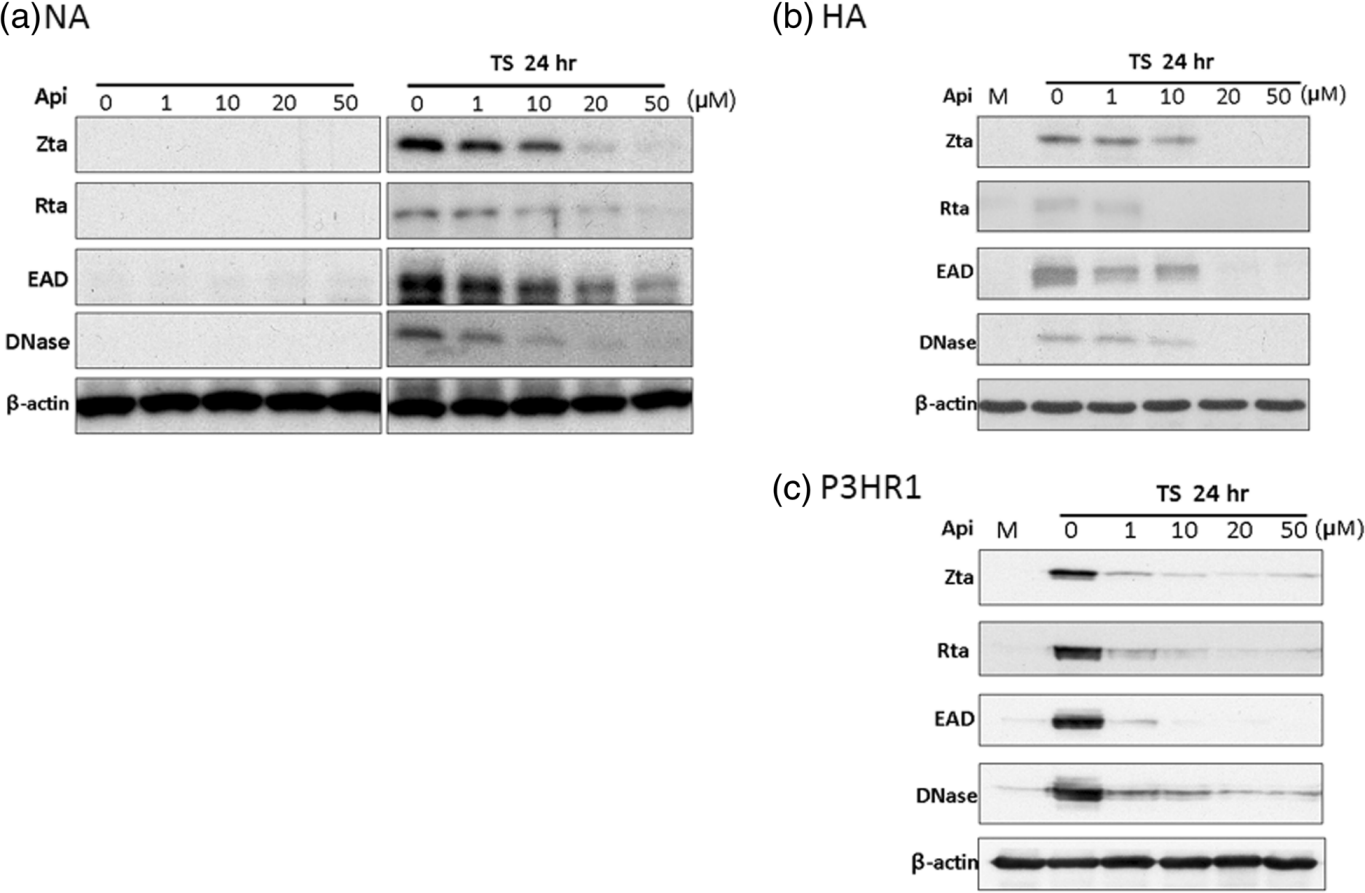
Apigenin inhibits the expression of EBV lytic proteins in EBV-positive Cells. (**a**) Epithelial NA cells were tested to detect the expression of EBV lytic proteins. Left panel: for detection of enhancement of reactivation, various concentrations of apigenin were added to the cells for 25 h, and then cell lysates were collected for western blotting. Right panel: for detection of inhibition of reactivation, the cells were pre-treated with apigenin for 1 h, then TPA + SB co-treatment was used for EBV induction. After 24 h of incubation, the cell lysates were analyzed by western blotting with antibodies against EBV Zta, Rta, EAD, DNase and β-actin. (**b**) Epithelial HA cells and (**c**) Burkitt’s lymphoma P3HR1 cells were tested for detection of inhibition of EBV reactivation using a similar procedure

We next sought to determine whether apigenin has the ability to induce or inhibit EBV reactivation. Two EBV positive-NPC cell lines, NA and HA, were used for these experiments. After EBV reactivation, the immediate-early genes Zta and Rta are expressed, followed by numerous early and late proteins and, subsequently, the release of infectious virions. To determine whether apigenin can induce EBV reactivation, NA cells were treated with apigenin for 24 h and cell lysates were collected for the detection of EBV lytic proteins Zta, Rta, EAD and DNase by western blot analysis. As shown in the left panel of Figure 2a, apigenin did not induce any lytic protein expression in NA cells, suggesting apigenin cannot induce EBV reactivation. Next, we tried to determine whether apigenin inhibits EBV reactivation in NPC cells. For NPC cells, TPA + SB treatment is an effective way to activate EBV into the lytic stage and induce lytic protein expression [27]. Moreover, to achieve greater efficiency of apigenin treatment, plated cells were pre-treated with apigenin for 1 h prior to treatment with TPA (40 ng/ml) and SB (3 mM). After a further 24 h incubation, cell extracts were collected for detection of lytic proteins by western blot analysis. For the NA cells, the result showed that EBV could express lytic proteins normally, however, protein expression was gradually repressed following apigenin treatment, showing that apigenin has the ability to inhibit EBV reactivation (right panel, Figure 2a). To avoid the possibility of cell specificity, we used another EBV-positive NPC cell line, HA, to determine whether EBV reactivation can be inhibited by apigenin treatment. The result shown in Figure 2b reveals that administration of apigenin inhibited EBV lytic reactivation with a dose-dependent tendency in HA cells. The expression of lytic proteins was almost completely blocked with 20 and 50 μM apigenin treatment, suggesting a significant inhibitory effect (Figure 2b). In addition, because B cells constitute another natural host cell type of EBV, we investigated whether apigenin can inhibit EBV reactivation in B cells. The EBV-positive Burkitts’ lymphoma cell line P3HR1 was tested using the procedure described above. As shown in Figure 2c, apigenin treatment also inhibited EBV lytic protein in P3HR1 cells. These results revealed that apigenin has the capability to inhibit EBV lytic protein expression, no matter whether in epithelial or B cells.

### Immunofluorescence analysis of inhibition of the EBV lytic cycle by apigenin treatment

**Fig. 3:**
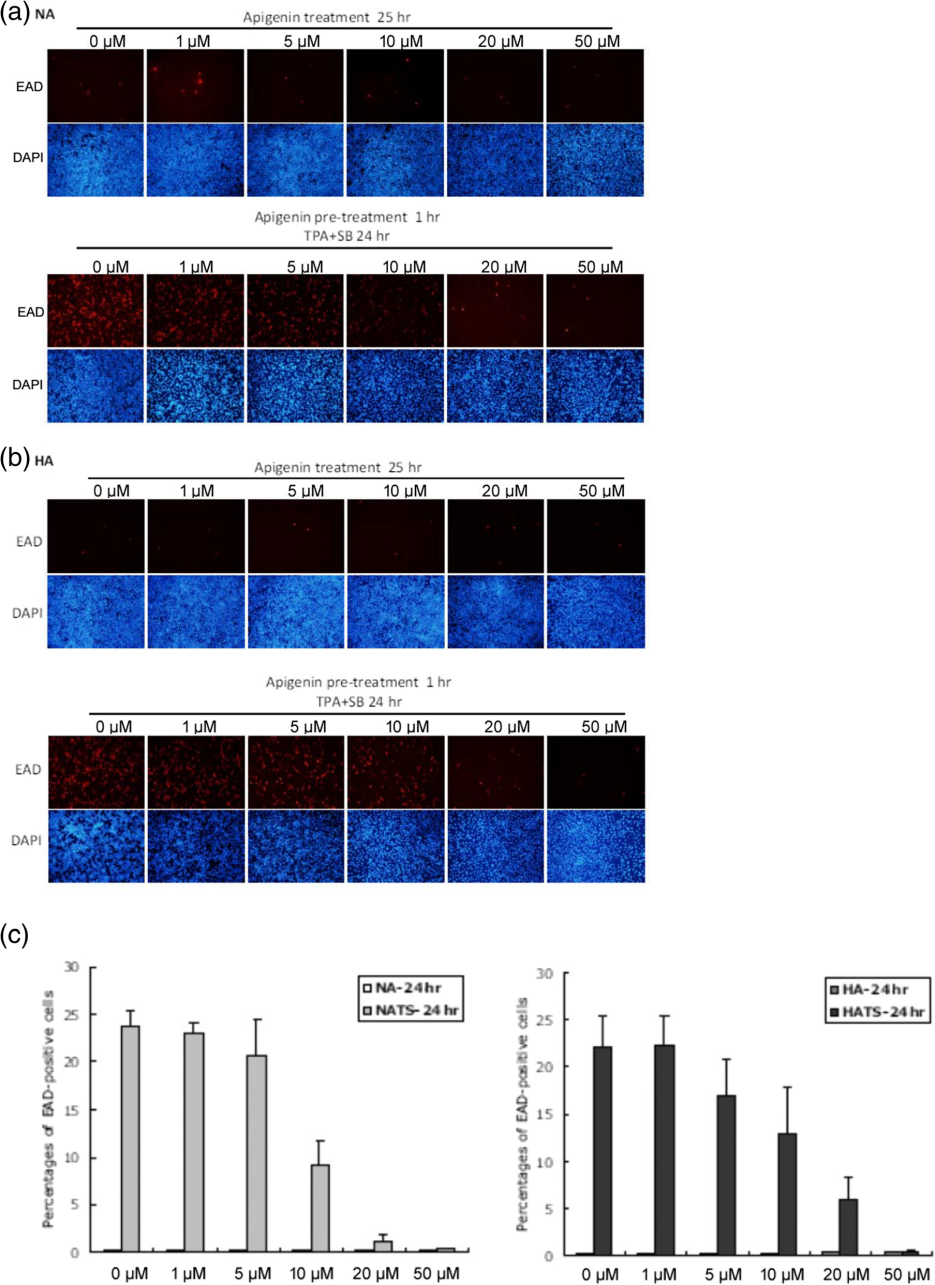
Apigenin decreases the populations of EAD-expressing cells, detected by immunofluorescence analysis NA (**a**) and HA (**b**) cells were processed for immunofluorescence analysis (IFA). For detection of inhibition of reactivation, the cells were pre-treated with various concentrations of apigenin for 1 h, then TPA + SB were added for EBV induction. After 24 h of incubation, the cells were analyzed by IFA with antibody against EBV EAD. (**c**) The percentages of EAD-expressing cells in each sample were calculated. The values are means ± SD from three separate experiments

Next, to confirm the inhibitory effect of apigenin, we examined the induction or inhibition of EBV lytic reactivation by detecting EAD expression using immunofluorescence analysis without or with TPA + SB induction. NA and HA cells were incubated with various concentrations of apigenin for 25 h. The results were that apigenin cannot induce EAD expression in NA and HA cells (Figure 3a and b, upper panels). For detection of inhibition of EBV reactivation, NA and HA cells were pre-treated with various concentrations of apigenin for 1 h, then EBV was activated by adding TPA + SB. After a further 24 h incubation, the immunofluorescence results revealed that the number of EAD-expressing cells decreased gradually with treatment of NA cells with increasing concentrations of apigenin (Figure 3a, lower panel). Apigenin repressed the numbers of EAD-expressing NA cells significantly at 10 μM, and blocked EAD expression completely at 20 ~ 50 μM. A similar result was observed for HA cells (Figure 3b, lower panel). Apigenin inhibited the numbers of EAD-expressing HA cells gradually in a dose-dependent manner (Figure 3b, lower panel). To analyze this inhibitory effect in more detail, we quantified the numbers of cells expressing EAD. As shown in Figure 3c, the numbers of EAD-expressing cells decreased gradually with apigenin treatment (Figure 3c). Taken together, the results of western blotting and immunofluorescence analysis, we conclude that apigenin can repress EBV lytic protein expressions, suggesting it has the ability to inhibit EBV reactivation.

### Inhibition of EBV virion production by apigenin

**Fig. 4:**
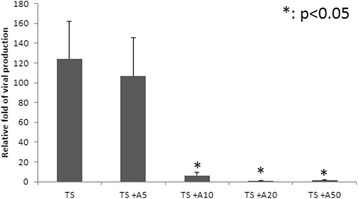
Apigenin inhibits virion production. After EBV reactivation, culture media containing released EBV particles were collected for qPCR analysis to detect the amount of EBV DNA in released EBV particles, as described in Methods. The relative EBV copy numbers were calculated. These are expressed as the relative folds to mock control. The values are means ± SD from three separate experiments

We have already shown that apigenin blocks the expression of EBV lytic proteins. A further question was whether apigenin can inhibit the EBV lytic cycle completely. NA cells were pre-treated with apigenin for 1 h, then TPA + SB was added. After incubation for 48 h, the supernatants were collected to measure the amounts of EBV virions. As shown in Figure 4, the production of EBV virions decreased gradually with increasing apigenin treatment. EBV virion production decreased significantly following treatment with 10 μM apigenin and was eliminated almost completely with 20 and 50 μM apigenin after induction (Figure 4). This result suggests that apigenin can inhibit not only EBV lytic protein expression but also virion production.

### Apigenin represses Zp and Rp activities following TPA + SB treatment and Zta/Rta induction

**Fig. 5:**
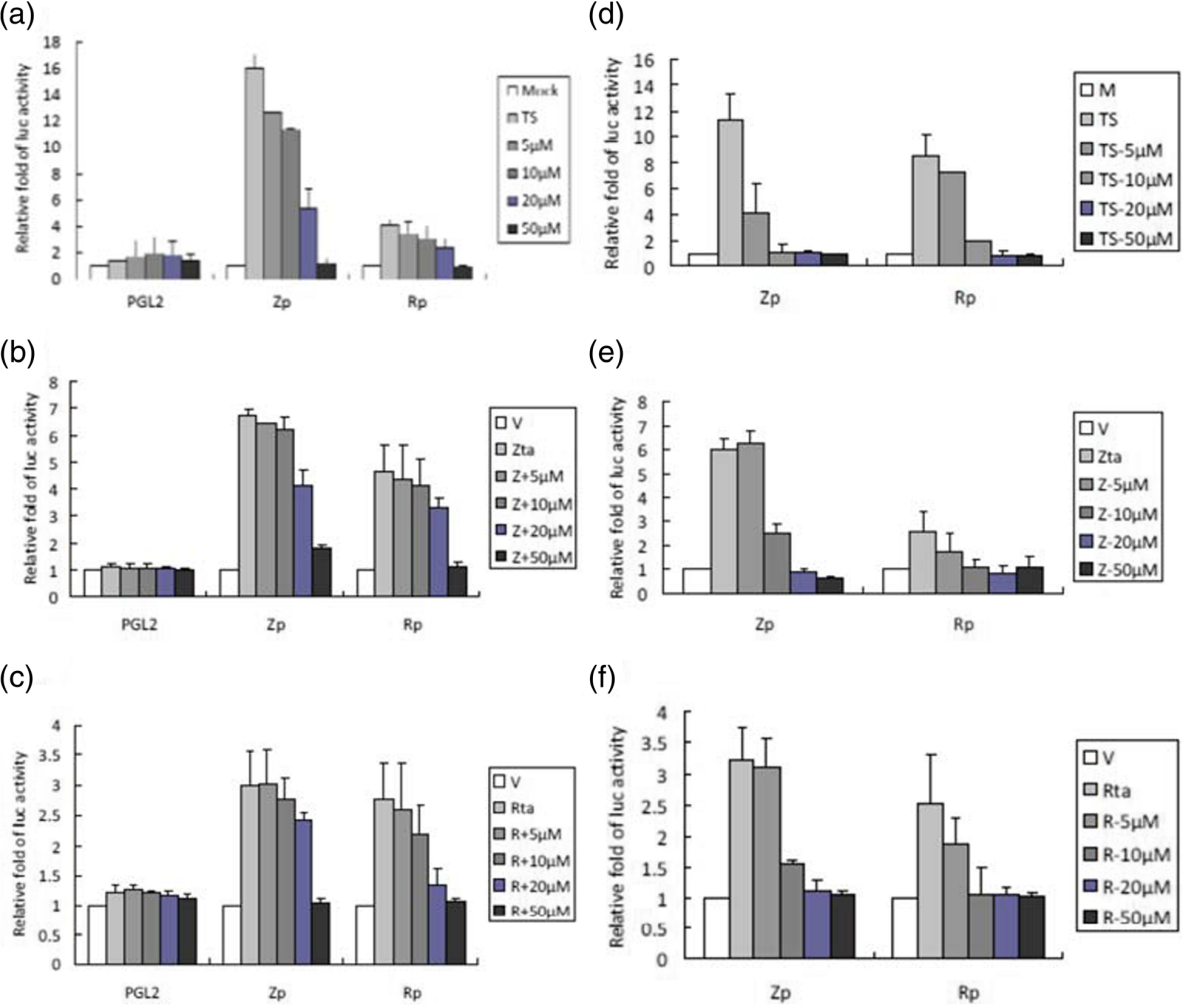
Apigenin represses Zp and Rp activities under different inductions. (**a**) Control plasmid PGL2, Zp, or Rp was transfected into NA cells. After 3 ~ 4 h of transfection, apigenin was added or not for pre-treatment for 1 h, and then TPA + SB were used to induce EBV into the lytic cycle. After induction for a further 24 h, lysates were collected for measurement of luciferase activity. Zp or Rp were co-transfected with (**b**) Zta-expressing and (**c**) Rta-expressing plasmids into NA cells for 3 h, then apigenin was added. After 24 h of transfection, luciferase activity was detected in the cell lysates. (**d**) Zp or Rp plasmid was transfected into TW01 cells. After 3 ~ 4 h of transfection, apigenin was added or not for pre-treatment for 1 h, and then TPA + SB was used to induce EBV reactivation. After induction for 24 h, cells lysates were produced for measurement of luciferase activity. Zp or Rp were co-transfected into TW01 cells with (**e**) Zta-expressing or (**f**) Rta-expressing plasmids. After 24 h of transfection, luciferase activity was detected in the cell lysates. The mean and standard deviation of each sample were calculated based on duplicates from three independent experiments

Zta and Rta are the two most important proteins that initiate the EBV lytic stage. To investigate further the inhibitory mechanism of action of apigenin, we sought to determine whether apigenin affects Zta and Rta promoter activities (Zp and Rp) under different stimuli. For chemical induction, luciferase reporters containing Zp and Rp were transfected into NA cells for 3 h. The cells were pre-treated with various amounts of apigenin for 1 h, TPA + SB were added for a further 24 h to induce EBV reactivation. Luciferase activity was determined subsequently as described in Materials and Methods. As expected, for the positive control, the luciferase activities of Zp and Rp both increased significantly after TPA + SB induction, compared to the mock-transfected control (Figure 5a, 0 μM). Meanwhile, the luciferase activities of Zp and Rp were gradually repressed by increasing concentrations of apigenin (Figure 5a). Co-treatment with TPA + SB and 50 μM apigenin reduced the activities of Zp and Rp to the mock-transfected control level. The result of NA cells transfected with the empty vector PGL2 showed that all values were at background levels (Figure 5a).

In addition to inhibiting the induction by chemicals, we tried to use another method of EBV induction to elucidate further the possible mechanism by which apigenin inhibits the EBV lytic cycle. Because the Zta response element (ZRE) and Rta response element (RRE) are present within Zp and Rp, respectively, Zp and Rp have been reported to be transactivated by the Zta and Rta proteins [34]. Therefore, Zta- and Rta-expressing plasmids were cotransfected with Zp or Rp into NA cells for 3 h, followed by apigenin treatment for 24 h. As expected, Zta expression in NA cells induced the promoter activities of Zp and Rp without apigenin treatment (Figure 5b, Zp & Rp, 0 μM). After co-treatment with apigenin, the promoter activities of Zp and Rp were reduced in a dose-dependent manner (Figure 5b, Zp & Rp). In addition, Rta expression also induced Zp and Rp activities, however, these were repressed by apigenin treatment, compared to that of the control reporter PGL2 (Figure 5c). In addition, more inducers were tested to confirm these phenomena. The results indicated that apigenin also inhibits Zp activity in a dose-dependent manner following induction by various HDAC inhibitors [35, 36] (**Additional file** 2).

To avoid the effect of EBV activity in NA cells, we used the parental TW01 cells to detect the inhibitory effects of apigenin, in a similar manner to that described above. As shown in Figure 5d, TPA + SB treatment induced Zp and Rp significantly and this increase was inhibited by addition of apigenin. Similarly, increased Zp and Rp activities induced by Zta and Rta expression also were repressed by apigenin treatment of TW01 cells (Figures 5e and f).

Taken together, these results indicated that apigenin inhibits the EBV lytic cycle initiation by repressing IE promoter activities, stimulated by various inducers.

### The ZIIIA/B, Z1D and ZII elements of Zp are important in inhibition of apigenin

**Fig. 6:**
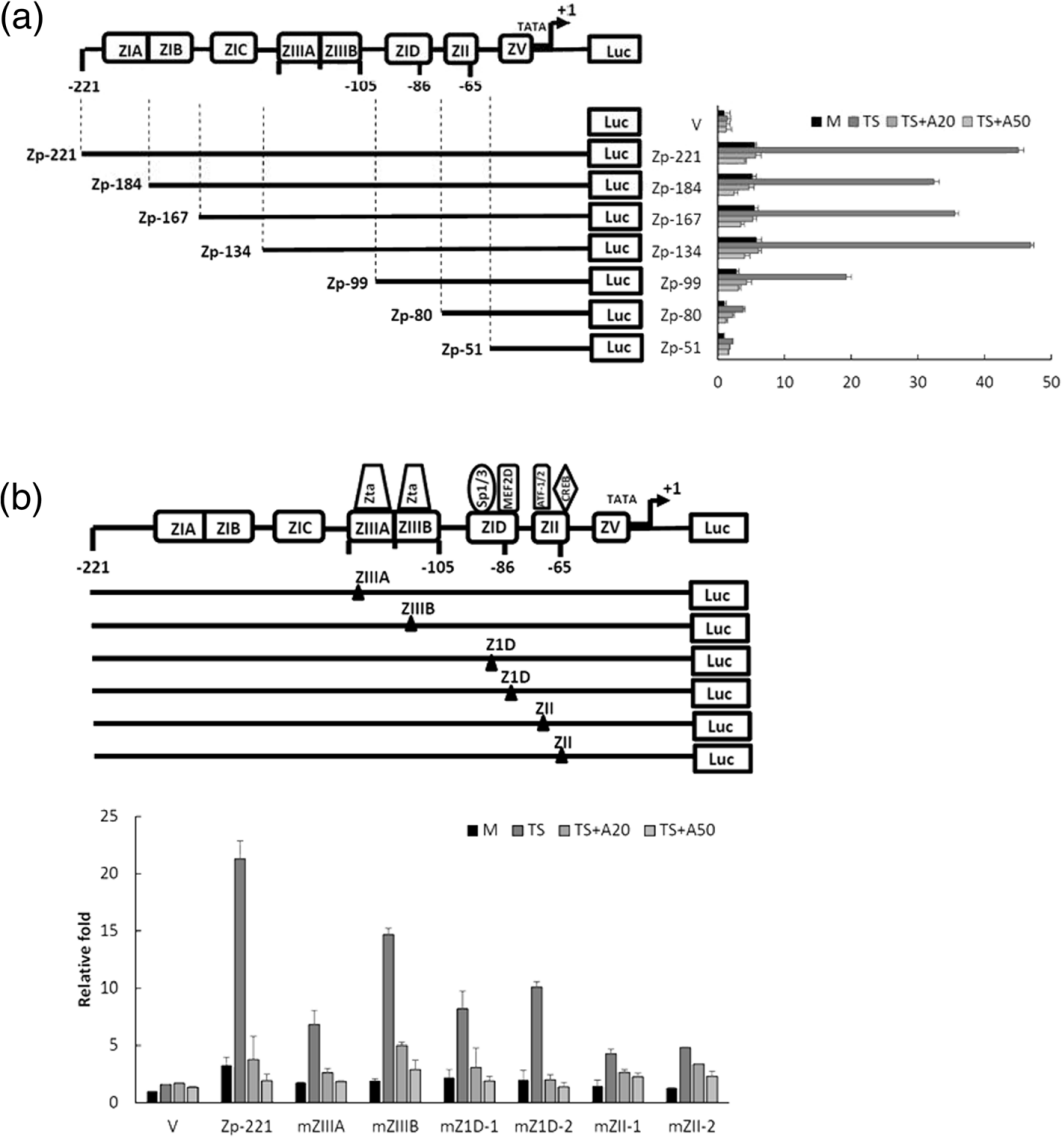
Identification of the response element of Zp required for apigenin inhibition of EBV. (**a**) A schematic diagram of the −221 to +1 region of Zp that drives the luciferase gene in the reporter plasmid (left panel). For the analysis of activities of Zp deletion mutants in response to apigenin plus TPA + SB induction. TW01 cells were transfected with these deletion mutants. Three hours after transfection, the cells were pre-treated with apigenin for 1 h and TPA + SB were added to activate EBV (right panel). The relative luciferase activities were calculated as described in Methods. (**b**) A schematic diagram of the indicated domain structure and cellular factor binding sites on Zp (−221 to +1). The relevant mutated sites are indicated by black triangle (▲) on the diagram (upper panel). For analysis of luciferase activities of Zp mutants upon apigenin plus TPA + SB treatment, TW01 cells were transfected with the mutants indicated for 3 h and pre-treated with apigenin, then TPA + SB were added for 24 h to activate EBV (lower panel). The luciferase activities were detected and were calculated as described in Methods. The mean and standard deviation of each sample were calculated in duplicate from at least two independent experiments

Because Zta is the first gene expressed after initiation of the EBV lytic cycle, we tried to determine what elements of Zp are essential for apigenin inhibition. For this purpose, we mapped the response domains of Zp required for apigenin inhibition. A series of 5’-deletion Zp constructs spanning from −221 to +12 region of Zp was transfected into TW01 cells for 3 h (Figure 6a, left panel). The cells were then pre-treated with 20 and 50 μM apigenin for 1 h, then TPA + SB was added. After treatment with TPA + SB for a further 24 h, the cell extracts were collected subsequently for detection of luciferase activity. For the TPA + SB - treated group (TS), the activities of serial deletion constructs from −221 to −134 were not affected, while deletion construct Zp-99 abolished a half of the Zp-221 activity, moreover, Zp-80 and Zp-51 retained little activity compared to vector control with mock treatment (Figure 6a, right panel). In addition, treatment with 20 and 50 μM apigenin repressed Zp activity in constructs Zp-221 to Zp-134 (Figure 6a, right panel), which showed a similar inhibition fold compared to that of each individual TS group (Figure 6a, right panel). However, the inhibition was gradually compromised in the Zp-99 to Zp-51 constructs (Figure 6a, right panel), suggesting the responsive element for apigenin inhibition of Zp induction is within the region −134 to −51.

There are three major domains located within the region −134 to −51, ZIIIA/B, Z1D and ZII (Figure 6a, left panel). ZIIIA/B is a regulatory region of Zp containing several Zta-binding-elements (ZREs). Z1D is an important domain involving Sp1/Sp3 and MEF2D regulation. The ZII region has been found to be an essential element for Zp activation and contains several transcriptional factor binding sites, including ATF-1, ATF-2 and CREB. To determine which regulatory factor was important for apigenin, various mutants of Zp were generated within the ZIIIA, ZIIIB, Z1D and ZII domains for luciferase analysis. TW01 cells were transfected with vector control (pGL2) wild-type Zp (Zp-221) and six mutants (mZIIIA, mZIIIB, mZ1D-1, mZ1D-2, ZII-1 and ZII-2). As shown in Figure 6b, the inhibition of Zp activity caused by apigenin was compromised significantly for mZIIIA, mZIIIB, mZ1D-1, mZII-1 and mZII-2, while activity of mZ1D-2 maintained the similar level as the wild-type control (upper and lower panels).

In summary, the ZIIIA/B, Z1D and ZII elements of Zp are important for Zp inhibition by apigenin, suggesting that the corresponding transcription factors may participate in Zp inhibition by apigenin.

## Discussion

EBV infection is prevalent worldwide and has been strongly associated with several human malignancies. Exploring new drugs with greater efficacy and less cytotoxicity is an important approach to conquering this threat. For this purpose, apigenin was identified by screening and we found it to have an effective inhibitory effect against EBV reactivation. Apigenin exerted great inhibition of EBV lytic protein expression, not only in epithelial cells but also in B cells (Figures 1 and 2). It also repressed the numbers of EBV-reactivating cells (Figure 3) and inhibited EBV production (Figure 4). Further study indicated that apigenin repressed the promoter activities of two IE genes, Zp and Rp, following chemical and Zta/Rta induction (Figures 5). To determine which elements of Zp were important for inhibition, we demonstrated that the ZIIIA/B, Z1D and ZII domains were involved in Zp inhibition by apigenin. These results demonstrate apigenin can inhibit EBV reactivation by repressing the promoter activities of two IE genes.

Anti-EBV compounds can be divided into two major categories: (1) those that interfere with virus-encoded enzymes important for virus production, e.g. ACV, GCV and BAY 57–1293 [37], and (2) those that interfere with the cellular processes required for virus production, e.g. the CDK inhibitor roscovitine [38]. The compounds belonging to the former category target selectively a specific enzyme and several disadvantages have emerged, such as viral resistance and a narrow spectrum. To avoid this limitation, compounds targeting cellular signaling pathways were developed as new anti-EBV drugs. As a large family of natural compounds, the flavonoids have been less well studied for their anti-virus functions. Among them, apigenin was reported to inhibit enterovirus-71 infection by disrupting viral RNA associated factors [39] and it also inhibits hepatitis C virus replication by decreasing microRNA122 [40] and restricts FMDV infection by inhibiting viral translational activity [41]. Moreover, although several flavonoids have been reported to have anti-EBV activity, these studies did not explore their inhibitory mechanisms and most such studies were made using the B cell system [42, 43].

Compared with conventional agents, as an anti-viral agent, apigenin has disadvantages such as lower specificity; however, it has many other attractive benefits, such as low cost, availability, safety and convenience. In addition to apigenin, curcumin has been shown to have an inhibitory effect on Zp to block the EBV lytic cycle in B cells [22]. Using a reporter assay, retinoic acid was also found to have potent anti-EBV activity [20]. Compared to some other natural compounds affecting only Zp [22] or Rp [44], apigenin is able to interfere with both Zp and Rp activities, suggesting it may have a broader spectrum for application.

Because Zp is the first promoter to be activated during the EBV lytic cycle, how apigenin inhibits Zp is a key issue for further study and application. There are eight domains located within Zp, and various cellular factors are involved in these domains. Among them, ZIIIA/B, Z1D, and ZII may play regulatory roles in apigenin inhibition (Figure 6). ZIIIA and ZIIIB have several Zta-binding motifs in these regions, and are responsible for TPA-induced Zp activation [31]. For Z1D, binding domains of Sp1/Sp3 and MEF2D have been identified in this region and their binding has also been shown to play important roles in EBV induction [30, 45–47]. For the ZII region, it was found that cellular proteins ATF-1/2 are predominant factors for Zp activation in this region, and the CREB/AP-1 family proteins are involved in it as well [32]. Based on our finding, Zp inhibition by apigenin may be through interacting with Sp1/3, ATF-1/2 and CREB (Figure 6). After reviewing the literature, flavonoids have been found to have abilities to suppress Sp1 activity [48], interact with ATF-1/2 [49–51] and change the phosphorylation status of CREB [52]. From our results, we can postulate reasonably that apigenin inhibits Zp induction likely through a complex process, which may involve a combination of the Sp1/Sp3, ATF-1/2 and CREB pathways.

Another two possible mechanisms of apigenin inhibition are blockage of the src, MAPK/p38 kinase pathway and ROS generation. Cellular signaling pathways such as protein kinase C, src or p38 kinase are necessary for EBV reactivation [53, 54]. Recently, we found that ROS plays a crucial role in EBV reactivation following N-methyl-N'-nitro-N-nitrosoguanidine (MNNG) treatment [55]. Apigenin can block the src, PKC and p38 signaling pathways [56, 57]. It is also shown to be a strong ROS scavenger [58]. It is reasonable to propose that apigenin acts to inhibit EBV reactivation through some of these mechanisms. Further studies are in progress.

Development of cancer treatments to improve survival and the quality of life of cancer patients is an important issue, especially during the past three decades. The major treatments for cancer include chemotherapy, surgery, radiation and immunotherapy. Recently, the concept, so-called “antimicrobial adjuvant therapy”, has been proposed to treat virus-related malignancies. For treatment of EBV-associated cancer, induction-lytic or anti-EBV strategies are studied for the treatment of EBV-related malignancies. The former strategy is more effective on regression of tumors, however, the escaping tumor cells may become more malignant because the expression of EBV lytic proteins in an abortive lytic state can cause genome instability and then increased tumorigenesis. In addition, although an anti-EBV strategy is weak on tumor remission, the patients have less risk from escaping EBV-lytic cells. In other words, the anti-EBV strategy is more suitable for prevention. In recent years, the concept of chemoprevention has been growing rapidly in oncology. This focuses on the prevention of cancer using natural or synthetic compounds. Apigenin is a natural plant flavone and it was first shown to have chemopreventive properties by Birt et al. [59]. Until now, apigenin has been found to have several anti-cancer functions: anti-oxidant, anti-mutagenic, anti-proliferative, anti-carcinogenic and anti-progression properties [24]. We believe that through these anti-cancer properties, combined with the anti-EBV effect, apigenin will provide a more profound benefit in chemoprevention and therapy of EBV-related malignancies.

Compared to the numerous studies focused on anticancer and antioxidation, the antiviral activities of apigenin have been less well studied. In fact, there are some studies suggesting that apigenin has anti-viral activity against other viruses [40, 60]. It is worthy of further study further to determine whether apigenin has an inhibitory effect on various other families of viruses.

## Conclusion

In this study, we found that the flavonoid apigenin inhibits EBV reactivation by repressing EBV IE promoter Zp and Rp activities. This finding may provide useful information for drug development and apigenin may be an alternative choice for therapy and prevention of EBV-related malignancies.

## Additional files

Additional file 1: The cytotoxicity of apigenin plus TPA + SB to epithelial cells and B cells (a) NA, (b) HA and (c) P3HR1 cell lines were pre-treated with apigenin for 1 h and then TPA + SB were added for 24 h. Cell viability was determined by WST-1 assay, as described in Methods. The values are means ± SD from at least two separate experiments. CC_50_ values also were calculated and are given at the top of each group. (TIF 146 kb)
Additional file 2: Apigenin represses Zp activity upon HDACi induction Control plasmid PGL2 or Zp was transfected into NA cells. Three hours after of transfection, apigenin was added or not for pre-treatment for 1 h and then various HDAC inhibitors, including (a) SB (3 mM); (b) TSA (5 μM) [30]; (c) SAHA (10 μM) [35] and (d) romidepsin (10 nM) [36], were used to induce EBV into the lytic cycle. After induction for a further 24 h, lysates were collected for measurement of luciferase activity. The mean and standard deviation of each sample were calculated in duplicate from at least two independent experiments. (TIF 100 kb)

## Abbreviations

ACV: Acyclovir
BL: Burkitt’s lymphoma
CC_50_: Cytotoxicity concentration
GCV: Ganciclovir
IE proteins: Immediate early protein
MNNG: N-methyl-N’-nitro-N-nitrosoguanidine
NPC: Nasopharyngeal carcinoma
SB: Sodium butyrate
TPA: 12-o-tetradecanoyl-phorbol-1,3-acetate

## References

[1] Rickinson AB, Kieff E, Knipe DM, Howley PM (2001). Epstein-Barr virus. Field’s Virology.

[2] Henle W, Henle G (1977). Evidence for an etiologic relation of the Epstein-Barr virus to human malignancies. The Laryngoscope.

[3] Ling W, Cao SM, Huang QH, Li YH, Deng MQ (2009). Prognostic implication of pretreatment titer of serum immunoglobulin A against Epstein-Barr virus capsid antigen in nasopharyngeal carcinoma patients in Sihui, Guangdong. Ai Zheng.

[4] Asito AS, Piriou E, Odada PS, Fiore N, Middeldorp JM, Long C, Dutta S, Lanar DE, Jura WG, Ouma C, Otieno JA, Moormann AM, Rochford R (2010). Elevated anti-Zta IgG levels and EBV viral load are associated with site of tumor presentation in endemic Burkitt’s lymphoma patients: a case control study. Infect Agent Cancer.

[5] Chen JY, Hwang LY, Beasley RP, Chien CS, Yang CS (1985). Antibody response to Epstein-Barr-virus-specific DNase in 13 patients with nasopharyngeal carcinoma in Taiwan: a retrospective study. Journal of medical virology.

[6] Chien YC, Chen JY, Liu MY, Yang HI, Hsu MM, Chen CJ, Yang CS (2001). Serologic markers of Epstein-Barr virus infection and nasopharyngeal carcinoma in Taiwanese men. N Engl J Med.

[7] Zeng Y, Zhang LG, Wu YC, Huang YS, Huang NQ, Li JY, Wang YB, Jiang MK, Fang Z, Meng NN (1985). Prospective studies on nasopharyngeal carcinoma in Epstein-Barr virus IgA/VCA antibody-positive persons in Wuzhou City, China. International journal of cancer.

[8] Cabras G, Decaussin G, Zeng Y, Djennaoui D, Melouli H, Broully P, Bouguermouh AM, Ooka T (2005). Epstein-Barr virus encoded BALF1 gene is transcribed in Burkitt’s lymphoma cell lines and in nasopharyngeal carcinoma’s biopsies. J Clin Virol.

[9] Luka J, Deeb ZE, Hartmann DP, Jenson B, Pearson GR (1988). Detection of antigens associated with Epstein-Barr virus replication in extracts from biopsy specimens of nasopharyngeal carcinomas. Journal of the National Cancer Institute.

[10] Zhang JX, Chen HL, Zong YS, Chan KH, Nicholls J, Middeldorp JM, Sham JS, Griffin BE, Ng MH (1998). Epstein-Barr virus expression within keratinizing nasopharyngeal carcinoma. Journal of medical virology.

[11] Fang CY, Lee CH, Wu CC, Chang YT, Yu SL, Chou SP, Huang PT, Chen CL, Hou JW, Chang Y, Tsai CH, Takada K, Chen JY (2009). Recurrent chemical reactivations of EBV promotes genome instability and enhances tumor progression of nasopharyngeal carcinoma cells. International journal of cancer.

[12] Fang CY, Huang SY, Wu CC, Hsu HY, Chou SP, Tsai CH, Chang Y, Takada K, Chen JY (2012). The synergistic effect of chemical carcinogens enhances Epstein-Barr virus reactivation and tumor progression of nasopharyngeal carcinoma cells. PloS one.

[13] Wu CC, Liu MT, Chang YT, Fang CY, Chou SP, Liao HW, Kuo KL, Hsu SL, Chen YR, Wang PW, Chen YL, Chuang HY, Lee CH, Chen M, Wayne Chang WS, Chen JY (2010). Epstein-Barr virus DNase (BGLF5) induces genomic instability in human epithelial cells. Nucleic acids research.

[14] Chang YH, Lee CP, Su MT, Wang JT, Chen JY, Lin SF, Tsai CH, Hsieh MJ, Takada K, Chen MR (2012). Epstein-Barr virus BGLF4 kinase retards cellular S-phase progression and induces chromosomal abnormality. PloS one.

[15] Chiu SH, Wu CC, Fang CY, Yu SL, Hsu HY, Chow YH, Chen JY (2014). Epstein-Barr virus BALF3 mediates genomic instability and progressive malignancy in nasopharyngeal carcinoma. Oncotarget.

[16] Hong GK, Gulley ML, Feng WH, Delecluse HJ, Holley-Guthrie E, Kenney SC (2005). Epstein-Barr virus lytic infection contributes to lymphoproliferative disease in a SCID mouse model. Journal of virology.

[17] Hong GK, Kumar P, Wang L, Damania B, Gulley ML, Delecluse HJ, Polverini PJ, Kenney SC (2005). Epstein-Barr virus lytic infection is required for efficient production of the angiogenesis factor vascular endothelial growth factor in lymphoblastoid cell lines. Journal of virology.

[18] Lin JC, Nelson DJ, Lambe CU, Choi EI (1986). Metabolic activation of 9 ([2-hydroxy-1-(hydroxymethyl) ethoxy] methyl) guanine in human lymphoblastoid cell lines infected with Epstein-Barr virus. Journal of virology.

[19] Wang FZ, Roy D, Gershburg E, Whitehurst CB, Dittmer DP, Pagano JS (2009). Maribavir inhibits Epstein-Barr virus transcription in addition to viral DNA replication. Journal of virology.

[20] Sista ND, Pagano JS, Liao W, Kenney S (1993). Retinoic acid is a negative regulator of the Epstein-Barr virus protein (BZLF1) that mediates disruption of latent infection. Proceedings of the National Academy of Sciences of the United States of America.

[21] Chang LK, Wei TT, Chiu YF, Tung CP, Chuang JY, Hung SK, Li C, Liu ST (2003). Inhibition of Epstein-Barr virus lytic cycle by (−)-epigallocatechin gallate. Biochemical and biophysical research communications.

[22] Hergenhahn M, Soto U, Weninger A, Polack A, Hsu CH, Cheng AL, Rosl F (2002). The chemopreventive compound curcumin is an efficient inhibitor of Epstein-Barr virus BZLF1 transcription in Raji DR-LUC cells. Molecular carcinogenesis.

[23] Wu CC, Chuang HY, Lin CY, Chen YJ, Tsai WH, Fang CY, Huang SY, Chuang FY, Lin SF, Chang Y, Chen JY (2013). Inhibition of Epstein-Barr virus reactivation in nasopharyngeal carcinoma cells by dietary sulforaphane. Molecular carcinogenesis.

[24] Patel D, Shukla S, Gupta S (2007). Apigenin and cancer chemoprevention: progress, potential and promise (review). International journal of oncology.

[25] Tsai CH, Williams MV, Glaser R (1991). Characterization of two monoclonal antibodies to Epstein-Barr virus diffuse early antigen which react to two different epitopes and have different biological function. Journal of virological methods.

[26] Tsai CH, Liu MT, Chen MR, Lu J, Yang HL, Chen JY, Yang CS (1997). Characterization of monoclonal antibodies to the Zta and DNase proteins of Epstein-Barr virus. Journal of biomedical science.

[27] Chang Y, Tung CH, Huang YT, Lu J, Chen JY, Tsai CH (1999). Requirement for cell-to-cell contact in Epstein-Barr virus infection of nasopharyngeal carcinoma cells and keratinocytes. Journal of virology.

[28] Hinuma Y, Konn M, Yamaguchi J, Grace JT (1967). Replication of herpes-type virus in a Burkitt lymphoma cell line. Journal of virology.

[29] Chen YJ, Tsai WH, Chen YL, Ko YC, Chou SP, Chen JY, Lin SF (2011). Epstein-Barr virus (EBV) Rta-mediated EBV and Kaposi’s sarcoma-associated herpesvirus lytic reactivations in 293 cells. PloS one.

[30] Tsai PF, Lin SJ, Weng PL, Tsai SC, Lin JH, Chou YC, Tsai CH (2011). Interplay between PKCdelta and Sp1 on histone deacetylase inhibitor-mediated Epstein-Barr virus reactivation. Journal of virology.

[31] Flemington E, Speck SH (1990). Autoregulation of Epstein-Barr virus putative lytic switch gene BZLF1. Journal of virology.

[32] Liu P, Liu S, Speck SH (1998). Identification of a negative cis element within the ZII domain of the Epstein-Barr virus lytic switch BZLF1 gene promoter. Journal of virology.

[33] Chiang LC, Ng LT, Lin IC, Kuo PL, Lin CC (2006). Anti-proliferative effect of apigenin and its apoptotic induction in human Hep G2 cells. Cancer letters.

[34] Lieberman PM, Hardwick JM, Sample J, Hayward GS, Hayward SD (1990). The Zta transactivator involved in induction of lytic cycle gene expression in Epstein-Barr virus-infected lymphocytes binds to both AP-1 and ZRE sites in target promoter and enhancer regions. Journal of virology.

[35] Hui KF, Ho DN, Tsang CM, Middeldorp JM, Tsao GS, Chiang AK (2012). Activation of lytic cycle of Epstein-Barr virus by suberoylanilide hydroxamic acid leads to apoptosis and tumor growth suppression of nasopharyngeal carcinoma. International journal of cancer.

[36] Hui KF, Cheung AK, Choi CK, Yeung PL, Middeldorp JM, Lung ML, Tsao SW, Chiang AK (2016). Inhibition of class I histone deacetylases by romidepsin potently induces Epstein-Barr virus lytic cycle and mediates enhanced cell death with ganciclovir. International journal of cancer.

[37] Kleymann G, Fischer R, Betz UA, Hendrix M, Bender W, Schneider U, Handke G, Eckenberg P, Hewlett G, Pevzner V, Baumeister J, Weber O, Henninger K, Keldenich J, Jensen A, Kolb J, Bach U, Popp A, Maben J, Frappa I, Haebich D, Lockhoff O, Rubsamen-Waigmann H (2002). New helicase-primase inhibitors as drug candidates for the treatment of herpes simplex disease. Nature medicine.

[38] Kudoh A, Daikoku T, Sugaya Y, Isomura H, Fujita M, Kiyono T, Nishiyama Y, Tsurumi T (2004). Inhibition of S-phase cyclin-dependent kinase activity blocks expression of Epstein-Barr virus immediate-early and early genes, preventing viral lytic replication. Journal of virology.

[39] Zhang W, Qiao H, Lv Y, Wang J, Chen X, Hou Y, Tan R, Li E (2014). Apigenin inhibits enterovirus-71 infection by disrupting viral RNA association with trans-acting factors. PloS one.

[40] Shibata C, Ohno M, Otsuka M, Kishikawa T, Goto K, Muroyama R, Kato N, Yoshikawa T, Takata A, Koike K (2014). The flavonoid apigenin inhibits hepatitis C virus replication by decreasing mature microRNA122 levels. Virology.

[41] Qian S, Fan W, Qian P, Zhang D, Wei Y, Chen H, Li X (2015). Apigenin restricts FMDV infection and inhibits viral IRES driven translational activity. Viruses.

[42] Iwase Y, Takemura Y, Ju-ichi M, Ito C, Furukawa H, Kawaii S, Yano M, Mou XY, Takayasu J, Tokuda H, Nishino H (2000). Inhibitory effect of flavonoids from citrus plants on Epstein-Barr virus activation and two-stage carcinogenesis of skin tumors. Cancer letters.

[43] Iwase Y, Takemura Y, Ju-ichi M, Mukainaka T, Ichiishi E, Ito C, Furukawa H, Yano M, Tokuda H, Nishino H (2001). Inhibitory effect of flavonoid derivatives on Epstein-Barr virus activation and two-stage carcinogenesis of skin tumors. Cancer letters.

[44] Chang FR, Hsieh YC, Chang YF, Lee KH, Wu YC, Chang LK (2010). Inhibition of the Epstein-Barr virus lytic cycle by moronic acid. Antiviral research.

[45] Chen C, Li D, Guo N (2009). Regulation of cellular and viral protein expression by the Epstein-Barr virus transcriptional regulator Zta: implications for therapy of EBV associated tumors. Cancer biology & therapy.

[46] Chua HH, Chiu HY, Lin SJ, Weng PL, Lin JH, Wu SW, Tsai SC, Tsai CH (2012). p53 and Sp1 cooperate to regulate the expression of Epstein-Barr viral Zta protein. Journal of medical virology.

[47] Liu S, Liu P, Borras A, Chatila T, Speck SH (1997). Cyclosporin A-sensitive induction of the Epstein-Barr virus lytic switch is mediated via a novel pathway involving a MEF2 family member. EMBO J.

[48] Yuan H, Gong A, Young CY (2005). Involvement of transcription factor Sp1 in quercetin-mediated inhibitory effect on the androgen receptor in human prostate cancer cells. Carcinogenesis.

[49] Guo Z, Du X, Iacovitti L (1998). Regulation of tyrosine hydroxylase gene expression during transdifferentiation of striatal neurons: changes in transcription factors binding the AP-1 site. J Neurosci.

[50] Kole L, Giri B, Manna SK, Pal B, Ghosh S (2011). Biochanin-A, an isoflavon, showed anti-proliferative and anti-inflammatory activities through the inhibition of iNOS expression, p38-MAPK and ATF-2 phosphorylation and blocking NFkappaB nuclear translocation. Eur J Pharmacol.

[51] Oleaga C, Ciudad CJ, Noe V, Izquierdo-Pulido M (2012). Coffee polyphenols change the expression of STAT5B and ATF-2 modifying cyclin D1 levels in cancer cells. Oxid Med Cell Longev.

[52] Yang MH, Kim J, Khan IA, Walker LA, Khan SI (2014). Nonsteroidal anti-inflammatory drug activated gene-1 (NAG-1) modulators from natural products as anti-cancer agents. Life sciences.

[53] Yu X, McCarthy PJ, Lim HJ, Iempridee T, Kraus RJ, Gorlen DA, Mertz JE (2011). The ZIIR element of the Epstein-Barr virus BZLF1 promoter plays a central role in establishment and maintenance of viral latency. Journal of virology.

[54] Miller CL, Lee JH, Kieff E, Longnecker R (1994). An integral membrane protein (LMP2) blocks reactivation of Epstein-Barr virus from latency following surface immunoglobulin crosslinking. Proceedings of the National Academy of Sciences of the United States of America.

[55] Huang SY, Fang CY, Tsai CH, Chang Y, Takada K, Hsu TY, Chen JY (2014). N-methyl-N’-nitro-N-nitrosoguanidine induces and cooperates with 12-O-tetradecanoylphorbol-1,3-acetate/sodium butyrate to enhance Epstein-Barr virus reactivation and genome instability in nasopharyngeal carcinoma cells. Chemico-biological interactions.

[56] Byun S, Park J, Lee E, Lim S, Yu JG, Lee SJ, Chen H, Dong Z, Lee KW, Lee HJ (2013). Src kinase is a direct target of apigenin against UVB-induced skin inflammation. Carcinogenesis.

[57] Lin JK, Chen YC, Huang YT, Lin-Shiau SY (1997). Suppression of protein kinase C and nuclear oncogene expression as possible molecular mechanisms of cancer chemoprevention by apigenin and curcumin. Journal of cellular biochemistry.

[58] Lin CM, Chen CT, Lee HH, Lin JK (2002). Prevention of cellular ROS damage by isovitexin and related flavonoids. Planta medica.

[59] Birt DF, Walker B, Tibbels MG, Bresnick E (1986). Anti-mutagenesis and anti-promotion by apigenin, robinetin and indole-3-carbinol. Carcinogenesis.

[60] Critchfield JW, Butera ST, Folks TM (1996). Inhibition of HIV activation in latently infected cells by flavonoid compounds. AIDS research and human retroviruses.

