# Supplementary figures and images for: Inhibition of Epstein-Barr virus reactivation by the flavonoid apigenin
Source: J Biomed Sci. 2017 Jan 5;24:2. doi: 10.1186/s12929-016-0313-9 (PMC5217310; doi:10.1186/s12929-016-0313-9)

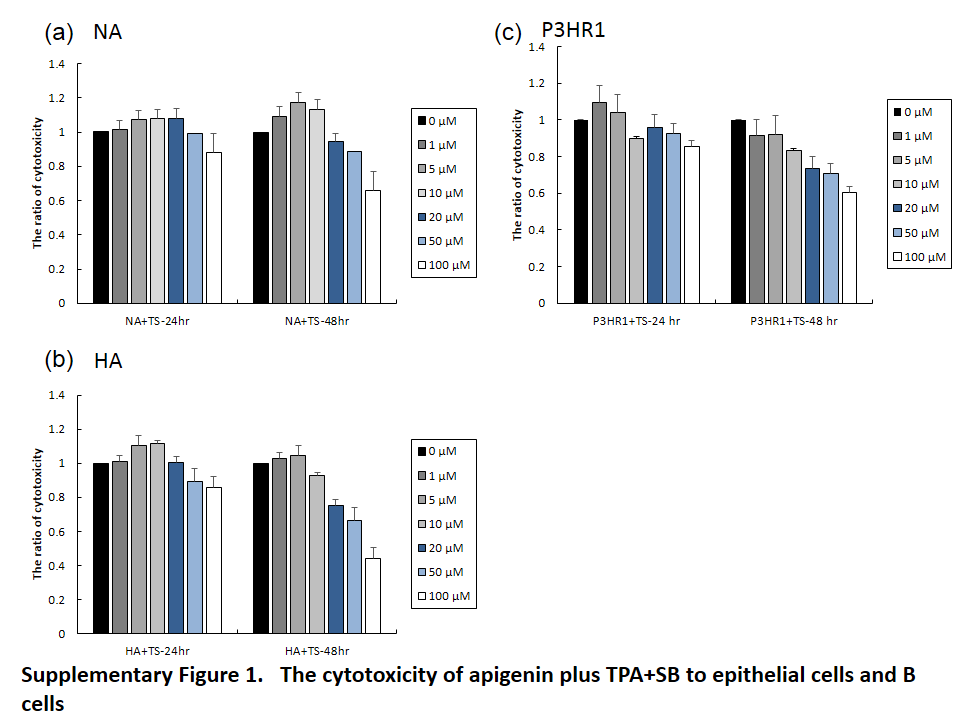

Supplement: Additional file 1: — The cytotoxicity of apigenin plus TPA + SB to epithelial cells and B cells (a) NA, (b) HA and (c) P3HR1 cell lines were pre-treated with apigenin for 1 h and then TPA + SB were added for 24 h. Cell viability was determined by WST-1 assay, as described in Methods. The values are means ± SD from at least two separate experiments. CC50 values also were calculated and are given at the top of each group. (TIF 146 kb) [file 12929_2016_313_MOESM1_ESM.tif]

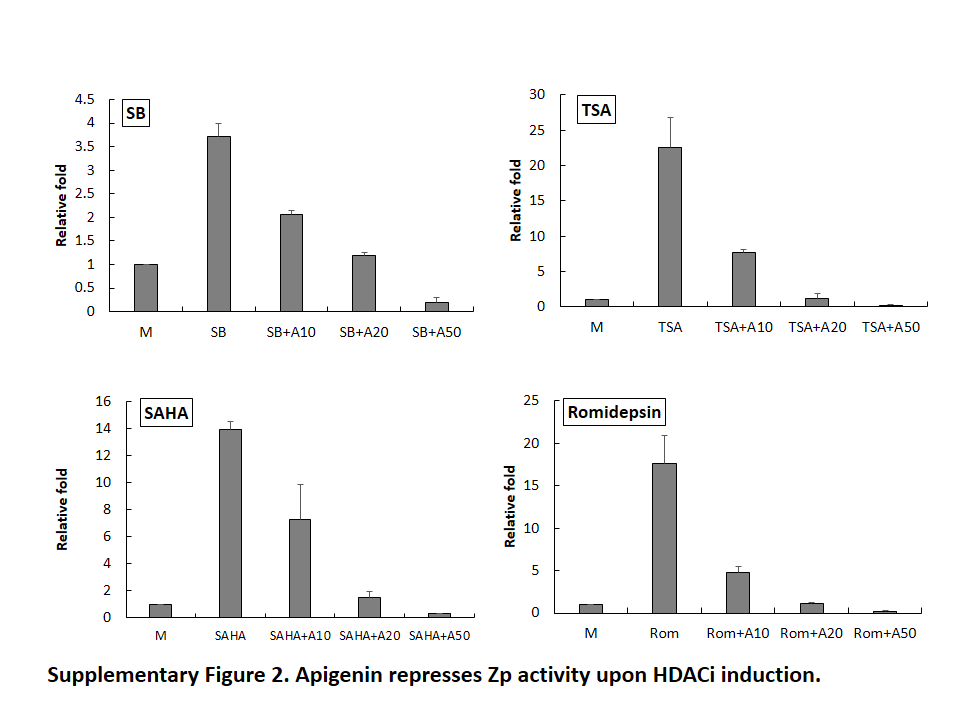

Supplement: Additional file 2: — Apigenin represses Zp activity upon HDACi induction Control plasmid PGL2 or Zp was transfected into NA cells. Three hours after of transfection, apigenin was added or not for pre-treatment for 1 h and then various HDAC inhibitors, including (a) SB (3 mM); (b) TSA (5 μM) [30]; (c) SAHA (10 μM) [35] and (d) romidepsin (10 nM) [36], were used to induce EBV into the lytic cycle. After induction for a further 24 h, lysates were collected for measurement of luciferase activity. The mean and standard deviation of each sample were calculated in duplicate from at least two independent experiments. (TIF 100 kb) [file 12929_2016_313_MOESM2_ESM.tif]
